# Composite grafts made of polycaprolactone fiber mats and oil-based calcium phosphate cement pastes for the reconstruction of cranial and maxillofacial defects

**DOI:** 10.1007/s00784-023-04932-4

**Published:** 2023-03-03

**Authors:** Andreas Fuchs, Michael Bartolf-Kopp, Hartmut Böhm, Anton Straub, Alexander C. Kübler, Christian Linz, Uwe Gbureck

**Affiliations:** 1 Department of Oral and Maxillofacial Plastic Surgery, University Hospital Würzburg, Pleicherwall 2, 97070 Würzburg, Germany; 2 Department for Functional Materials in Medicine and Dentistry, University Hospital Würzburg, Pleicherwall 2, 97070 Würzburg, Germany; 3 Department of Oral and Maxillofacial Plastic Surgery, Faculty of Medicine and University Hospital Cologne, Kerpener Straße 62, 50937 Cologne, Germany

**Keywords:** Fused deposition modeling, Polycaprolactone, Oil-based cement pastes, 3D printing, Composite graft

## Abstract

**Objectives:**

Synthetic bone substitutes which can be adapted preoperatively and patient specific may be helpful in various bony defects in the field of oral- and maxillofacial surgery. For this purpose, composite grafts made of self-setting and oil-based calcium phosphate cement (CPC) pastes, which were reinforced with 3D-printed polycaprolactone (PCL) fiber mats were manufactured.

**Materials and methods:**

Bone defect models were acquired using patient data from real defect situations of patients from our clinic. Using a mirror imaging technique, templates of the defect situation were fabricated via a commercially available 3D-printing system. The composite grafts were assembled layer by layer, aligned on top of these templates and fitted into the defect situation. Besides, PCL-reinforced CPC samples were evaluated regarding their structural and mechanical properties via X-ray diffraction (XRD), infrared (IR) spectroscopy, scanning electron microscopy (SEM), and 3-point-bending testing.

**Results:**

The process sequence including data acquisition, template fabrication, and manufacturing of patient specific implants proved to be accurate and uncomplicated. The individual implants consisting mainly of hydroxyapatite and tetracalcium phosphate displayed good processability and a high precision of fit. The mechanical properties of the CPC cements in terms of maximum force and stress load to material fatigue were not negatively affected by the PCL fiber reinforcement, whereas clinical handling properties increased remarkably.

**Conclusion:**

PCL fiber reinforcement of CPC cements enables the production of very freely modelable three-dimensional implants with adequate chemical and mechanical properties for bone replacement applications.

**Clinical relevance:**

The complex bone morphology in the region of the facial skull often poses a great challenge for a sufficient reconstruction of bony defects. A full-fledged bone replacement here often requires the replication of filigree three-dimensional structures partly without support from the surrounding tissue. With regard to this problem, the combination of smooth 3D-printed fiber mats and oil-based CPC pastes represents a promising method for fabricating patient specific degradable implants for the treatment of various craniofacial bone defects.

## Introduction

Bony defects represent a clinical problem that is very widespread in the field of maxillofacial surgery. These defects can occur in consequence of trauma (e.g. midface) [1, 2], chronic infections (e.g. osteomyelitis, chronic oroantral fistula) [3, 4], or iatrogenic (e.g. hemicraniectomy, tumor resection) [5, 6]. The treatment of such defect situations is oftentimes very difficult but necessary at the same time. The example of midface trauma in particular shows that the anatomical and functional complexity in this region often poses a major reconstructive challenge. This is already shown by the fact that there is no “one” midface fracture, but for functional reasons, a distinction is made between central midface fractures (naso-ethmoidal complex, frontal sinus, etmoid, orbita), Le Fort fractures, lateral midface fractures (zygomatic bone, zygomatic arch), and panfacial fractures [1]. Here, highly complex, three-dimensional bone structures meet, which can be loaded in different ways and are therefore difficult to reconstruct. Furthermore, the treatment of midface defects requires good knowledge regarding occlusion and the physiology of the eye, in addition to knowledge of the anatomical basis of the bony structures themselves due to their adjacency to the eye and the masticatory apparatus. With regard to the anatomical boundaries and neighboring regions, similar problems arise in the context of the therapy of chronic infections in the midface region. This is particularly true if the infection has progressed to the point of bone destruction in the form of an oro-antral fistula of varying degrees of severity [4]. In this case, the destruction of the facial or caudal border of the sinus is of primary importance, especially if the sinus maxillaris is involved. With regard to the reconstruction of the filigree bone structures, the preservation of the ocular function as well as the securing of the correct occlusion must always be taken into consideration in the therapy. Whereas in the viscerocranial region, as already mentioned several times, a very complex anatomy has to be considered, in the neurocranial region, at first glance, a less complex situation seems to be present. This may be true for the purely geometric conditions, but the sufficient reconstruction of the calvaria in bony defects in the area of the neurocranium ensures the function of the brain and therefore this reconstruction in particular is of decisive importance. As consequence of an insufficient reconstruction, repeated wound problems like dehiscence or infection may occur [7].

Reconstructive measures of the viscerocranium aim mostly at the reconstruction of the bone continuity, an aesthetic improvement, or the rehabilitation of the masticatory function. Within the neurocranium, the protection of vital structures, like the brain, plays an essential role [8]. In all aforementioned situations, the question has to be answered how the respective defects can be treated and which material should be used for this purpose. A definite answer to this is yet to be found.

Midfacial and periorbital reconstruction can, in principle and depending on its size, be performed with either (microvascular) autologous bone grafts or, far more commonly used synthetic bone replacement materials (e.g. patient specific implants) [9]. In this, especially for the reconstruction of the commonly diagnosed orbital floor fractures, several materials have been examined. These materials comprise, on the one hand non-resorbable synthetic bone grafts like silicone, bioactive glass, porous polyethylene, and titanium. On the other hand, there are also several resorbable synthetic materials, which are used in this indication like gelatin films, polyglycolic acid, and polydioxanone [10]. For the reconstruction of skull defects in terms of cranioplasty, titanium mesh is a widespread synthetic material for the reconstruction of skull defects of different extent [7]. Other synthetic bone replacement materials which are also established for means of cranioplasty are polymethylmethacrylate (PMMA), polyetheretherketone (PEEK), and polyethylene [11]. Furthermore, numerous other materials for cranioplasty have been introduced, such as PCL, hydroxyapatite, alumina ceramics [7, 12, 13]. Besides all aforementioned synthetic materials, autografts are also commonly used in skull reconstruction, however with the common disadvantages of donor-site morbidity and limited availability especially in cases of greater defects [11].

Besides the choice of a suitable bone reconstruction material itself, it has to be taken into consideration how the respective material can be processed. Here, the discrimination between prefabricated implants and implants which have to be modified intraoperatively plays an important role. As prefabricated implants are manufactured preoperatively mostly via CAD/CAM (computer aided design/computer aided manufacturing) on basis of patient-specific data (e.g. computed tomography), they promise a very good fitting which does not require extensive modification [14, 15]. Bone substitutes which have to be modified intraoperatively, such as cements, granulates, or sintered blocks, normally demand larger adjustments considering size and shape [16], which affects operating time negatively. Moreover, aesthetic and functional requirements can oftentimes only be met to a certain degree intraoperatively, as the defect situation cannot be assessed perfectly by the surgeon [17]. Implants which are manufactured patient specific by means of individual CAD/CAM life-size patient models, take a middle position between these two possibilities [18].

In this study,the possibility to fabricate composite bone substitutes consisting of 3D-printed fiber laminates enforced with calcium phosphate cement CPC for the treatment of craniofacial defects was evaluated. Fiber laminates were obtained by fused deposition modeling (FDM) and cements consisted of a self-setting, oil-based CPC paste. In previous works of our group, both components—PCL fiber scaffolds as well as oil-based cement pastes—on their own have proven excellent properties considering their use in maxillofacial applications [19–21]. In the present work, the attempt was made to combine their single qualities—amongst others—good clinical handling of the smooth fiber mats and high mechanical stability of CPCs—by making composite grafts and adapting them into real defect situations. Both components of the CPC-PCL fiber laminates were fused layer by layer and then aligned to different defect situations on patient specific individual models.

## Materials and methods

### Fiber mat fabrication

Custom-made devices were used to produce the fiber mats for CPC reinforcement by means of FDM. As a polymer, medical-grade polycaprolactone (PCL; Purac, PURASORB PC12, Corbion, Amsterdam, Netherlands) was used. FDM was performed with a commercially available printer (RegenHU Discovery, REGENHU, Villaz-St-Pierre, Switzerland) via a screw-driven extruder print head with a needle diameter of 200 µm. The process parameters of the FDM were a reservoir temperature of 89 °C, a needle temperature of 95 °C, a collector temperature of 25 °C, and a collector velocity of 10 mm/s. Fiber mats were designed box-structured by alternating the layer deposition via 0° and 90° layers (50 × 50 mm) in each direction with turning loops. One layer was deposited in each direction (0° and 90°) such that 2 fibers overlapped at intersections. Filament spacings between the PCL fibers amounted 1.5 mm.

### CPC paste preparation

Self-setting oil-based cement pastes were obtained by mixing the CPC raw powder with consisting of an equimolar mixture of tetracalcium phosphate (Ca_4_(PO_4_)_2_O, TTCP) and dicalcium phosphate anhydrous (CaHPO_4_, DCPA) with the addition of 1 wt.% sodium phosphate (Na_2_HPO_4_) and an oil-surfactant mixture as described elsewhere [21]. Mixing of cement powder and the oil phase was achieved by using a centrifugal mixer (ARV-310CE, Thinky Corporation) at 2000 rpm for 5 min.

### Laminate fabrication

The composite laminates were assembled according to a regime as it was reported previously by our working group [22]. For this, 1.5 g of the CPC pastes were smeared evenly on a weight paper in an approximate dimension of 50 × 50 mm. One PCL fiber mat was then added and pressed onto the wet cement paste. Afterwards, another layer of CPC paste was added and smeared evenly no top. This approach was repeated one more time until 2 layers of PCL fiber mats were covered with cement paste. Based on experiences of prior experiments, excess cement on MEW scaffolds was removed with aluminum foil after each layer. In a last step, CPC raw powder was sieved through a 300 µm sieve on top of the last cement paste layer in order to cover all oil residues. This was repeated on the other side of the laminate after separation from the weight paper on the backside of the laminate. Loose raw powder was removed by compressed air. Full-size (50 × 50 mm) laminates were used to fit clinical defect situations. For the mechanical evaluation, smaller test specimens with the dimensions 20 mm × 5 mm × 3 mm were cut out by hand.

### Data acquisition of the skull defects and computer-aided design of implant templates

In order to obtain realistic and clinically relevant examples for bony defects, radiological data of patients of the Department of Oral and Maxillofacial Plastic Surgery Wuerzburg were used. Datasets were acquired within regular treatment of the patients. This was approved by the ethics committee of the University of Wuerzburg (20200427 01). Defect situations were chosen according to a possible use of our CPC-PCL laminates mostly in non-load-bearing situations. Datasets of digital volumetric tomographies (DVT) or computed tomographies (CT) were transferred into a commercially available 3D planning software for image guided surgery (Mimics inPrint, Materialise, Leuven, Belgium). The virtual generation of the templates for implant manufacturing was performed using a mirror imaging procedure. Therefor the non-affected side was mirrored into the defect situation. Post-processing allowed to insert a virtual base/floor under the defects which enabled to customize the CPC-PCL laminates on top of them. The so acquired DICOM files were then translated into STL format and transferred to a commercially available 3D printing system (Stratasys Dimension Elite, Stratasys, Eden Prairie, USA). Furthermore, 3D models of the actual skull defect situation—as it was found clinically—were printed using the same aforementioned printing system from a production grade thermoplastic (ABSplus p430, Stratasys, Eden Prairie, USA) in order to examine fit and shape of the prospective bone implants.

### Implant fabrication

The composite laminates made out of CPC pastes and PCL scaffolds were then carefully adapted on top of the defect situations on the in the aforementioned way prepared CAD/CAM templates. In order to stabilize the CPC-PCL laminates, where necessary, support structures, which exceeded defect margins for about 5 mm in all directions, were integrated into the implants. Excess parts were cut out using scissors and scalpel. Afterwards, they were stored for 24 h at 37° C and > 90% humidity to initiate the setting reaction of the cement phase and finally cured in water for another 24 h at 37° C.

### Dimensional and physical analysis

In order to evaluate an adequate fit and the dimensional stability and precision of the composite laminates in the defect situations, they were inserted into the defect situations on the CAD/CAM control model. Afterwards surface matching and handling properties were assessed clinically. This was done in the first place by an optical assessment of laminate fitting in the margin areas and the restoration of the outer surface contour in comparison to the contralateral side. Furthermore, laminates were removed from the skull model and afterwards repositioned to evaluate their explicit repositioning-ability.

Scanning electron microscopy (SEM; CB 340, Zeiss, Oberkochen, Germany) was used to assess both the surface condition of the CPC-PCL laminates and the bond between the PCL fibers and the cements. Prior to SEM analysis, fiber laminates were washed four times in PBS and dehydrated in an ascending ethanol series (30–100%) at room temperature and finally dried with hexamethyldisilazane (Merck, Darmstadt, Germany). Finally, the samples were sputter coated with a thin conductive platinum layer (EM ACE600 Leica Microsystems, Wetzlar, Germany).

The composition of the CPC cements, initially and after 7 days of curing in a water bath, was investigated by X-ray diffraction (XRD). XRD patterns were recorded on a Bruker D8 Advance diffractometer with DAVINCI Design (Bruker, Karlsruhe, Germany) in a 2 Theta range from 20–40°, a step size of 0.02°, and a scan rate of 1.5 s/step. Qualitative evaluation of the respective diffraction patterns was performed using JCPDS references. Furthermore, Infrared (IR) spectroscopy was used to quantitatively evaluate the composition of CPC pastes before and after hardening for 7 days. FT-IR measurements were performed with a FT-IR spectrometer (Thermo Fisher Scientific Inc., Waltham, MA, USA) in attenuated total reflexion (ATR) mode with 16 scans each and a resolution of 4 cm^−1^.

For the 3-point-bending test, a ZwickRoell Z010 testing machine (ZwickRoell, Ulm, Germany) which measured the force using the 10 kN load cell was used. The test parameters were a pre-load of 0.1 N before the bolt is driven onto the specimen at a cross-head speed of 1 mm/min. The normalization of force to stress was then calculated over the dimensions of the test specimen. This way, six samples of PCL-fiber-reinforced cements and six samples of non-reinforced cements were tested. While the samples made from non-reinforced cement paste were prepared in silicone rubber molds measuring 20 mm × 5 mm × 3 mm, the PCL-reinforced samples were assembled manually and then cut into the respective shape and size comparable to the non-reinforced samples prior to hardening. The actual size was finally measured after hardening with a caliper and taken into account when calculation the maximum stress values. Statistical analysis of the mechanical properties was performed using Microsoft Excel 2013 (Microsoft, Redmond, USA) an independent two-sided *t-*test. A *p*-value of 0.05 or less was considered to demonstrate statistical significance.

## Results

### Laminate fabrication and fitting

**Fig. 1:**
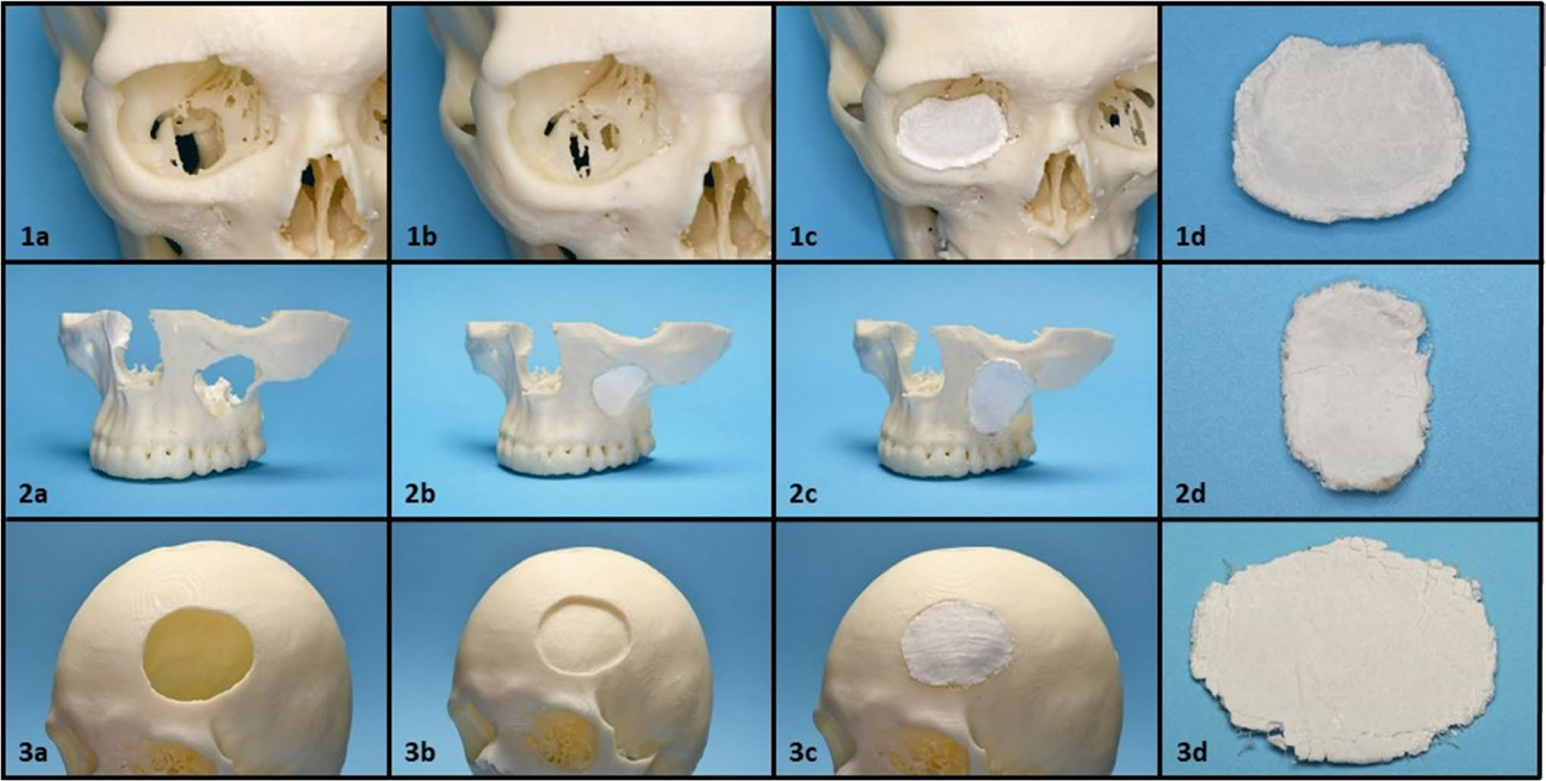
Application examples for fiber laminates in three different clinical defect situations. 1a–1c Fracture of the orbital floor, 2a–2c defect of the facial/lateral wall of the maxillary sinus, 3a–3c defect situation after a partial craniectomy. **a** Native defect situation as found in the respective patients, **b** defect situation virtually reconstructed by mirror imaging, **c** defect situation reconstructed with fiber laminates, **d** fiber laminates non in situ

In this study an indirect approach towards bone implant fabrication was chosen. For this, templates were built via CAD/CAM which proceeded very well, apart from minor initial problems in adjusting the mirrored bone structures into the defect areas. After removing small inhomogeneities and final tailoring for perfect fit, the fiber laminates were inserted into the different defect situations and placed over the defect situation in order to cover the defect (Fig. 1: 1c,2c,3c).

The contour of all implants matched the defect situation well in every observed cranial defect. Generally, simple convex or concave defect situations could be reconstructed with a higher accuracy (Fig. 1: 1c,3c). Complex defect geometries combining convex and concave structures caused most difficulties (Fig. 1: 2c). In the margin areas, smaller overlaps or gaps could be observed. After removing the overlaps with a scalpel, the fiber laminates could be easily placed into, or onto the defect situations without considerable interference.

In laminate fabrication itself, the initial smooth texture of the fiber laminates and their single components, enabled detailed matching of the yet flexible laminates onto the surfaces of the defect templates. After hardening by exposing to moisture, rigidity and stability increased and laminates were no longer deformable. Overall, handling properties of the not-hardened laminates were suitable. Compared with currently available membranes for dental use, fiber laminates were more stiff. Nevertheless, bending was possible to a certain degree. However, excessive bending led to a breakup of the cement phase, while the PCL scaffolds still held the fiber mats together (Fig. 1: 1d,2d,3d). The consistency of the laminates changed during the hardening process to that of gypsum. However, osteosynthesis with common osteosynthesis systems as they are in daily clinical use, could not be performed. Hardened fiber laminates showed a distinct brittleness and drilling of holes in the margin areas of the fiber laminates caused severe fragmentation of the cement, which made the stable insertion of osteosynthesis screws impossible.

### SEM imaging

**Fig. 2:**
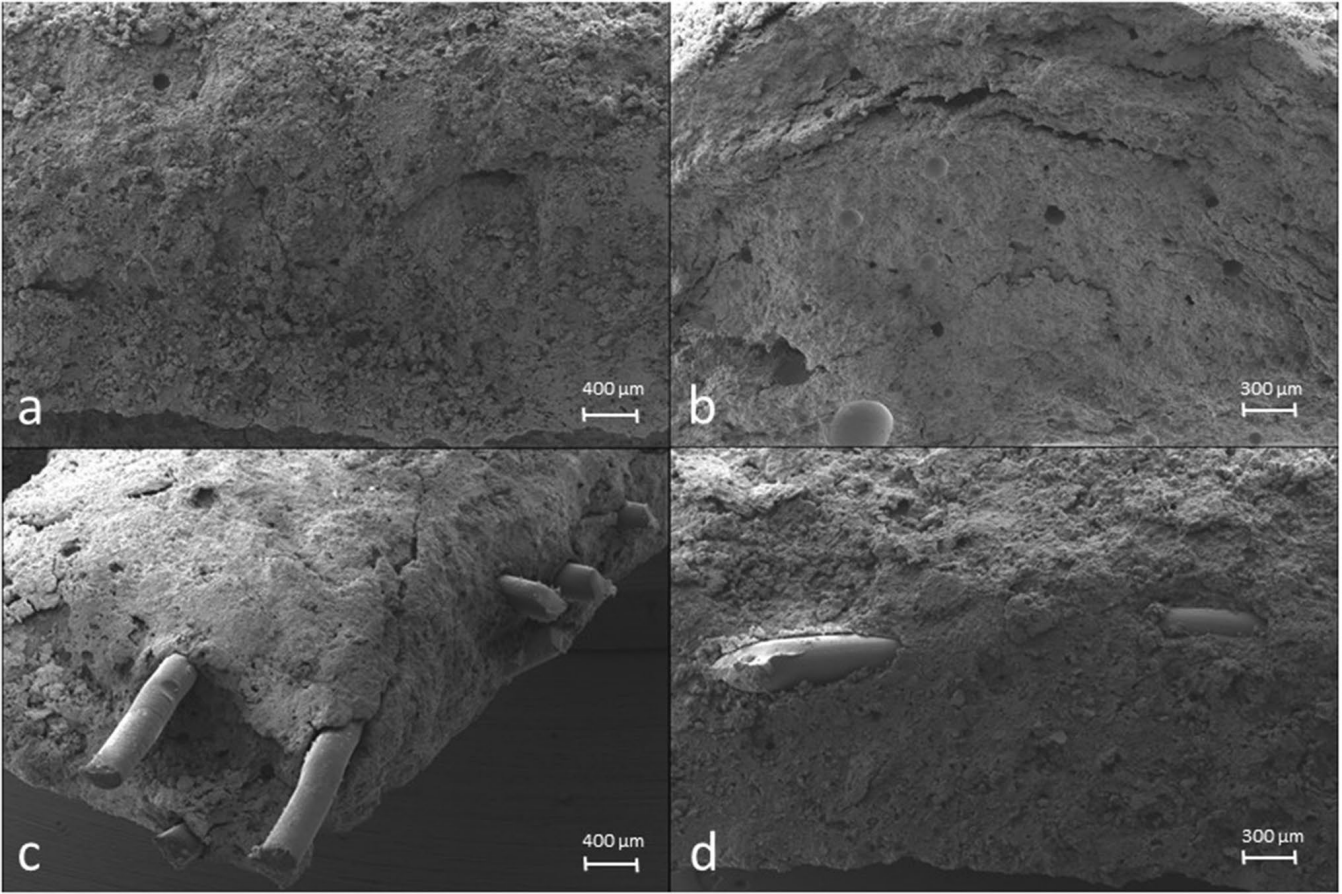
SEM images of not reinforced CPC samples (**a**, **b**) and PCL fiber reinforced samples (**c**, **d**) in lower (**a**, **c**) and higher magnification (**b**, **d**). All samples were hardened by storing at 37 °C > 90% humidity for 24 h followed by immersion in water for 7 days samples, followed by mechanical testing in 3-point-bending regime

The CPC displayed a relatively homogenous surface structure for both types of samples independently of their PCL reinforcement (Fig. 2). In some cases, smaller cracks on the surface could be observed, which did not trench the whole sample. Altogether, SEM imaging showed that PCL fibers were very well embedded into the CPC matrix of the reinforced fiber laminates. If at all, only small gaps could be observed at the interface of cement and polymeric fiber. Within this, fibers did not seem to be mechanically destroyed in the fabrication process.

### X-ray diffraction analysis

**Fig. 3:**
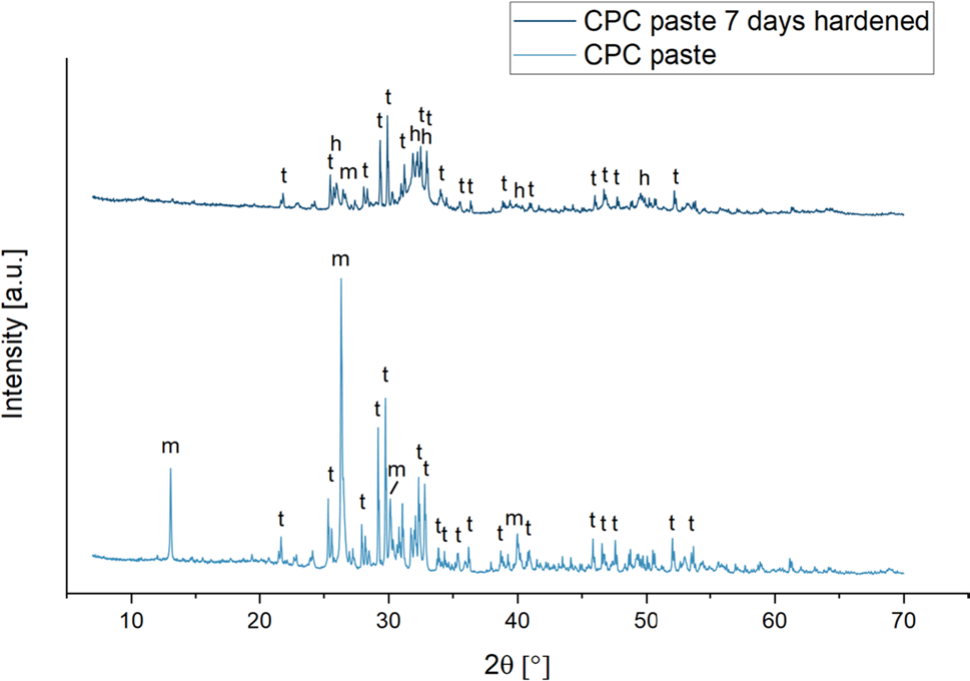
XRD evaluation of freshly prepared CPC pastes and after hardening for 7 days in water at 37 °C; m: monetite, h: hydroxyapatite, t: tetracalcium phosphate

XRD analysis revealed that the not hardened CPC consisted mainly of hydroxyapatite (6.1%), tetracalcium phosphate (48.7%) and monetite (45.2%). This ratio changed after 7 days of curing in a water bath: the proportion of hydroxyapatite increased to 59.3%, whereas the share of tetracalcium phosphate and monetite sank to 32.0% and 8.7% respectively (Fig. 3).

### Infrared spectroscopy analysis

**Fig. 4:**
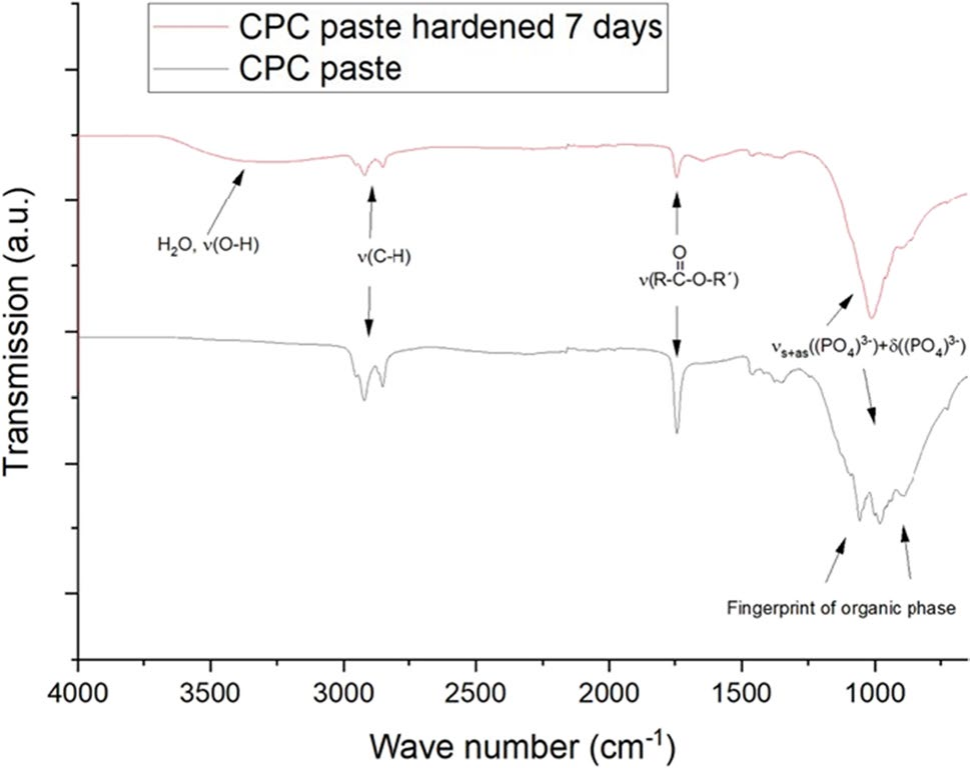
FT-IR spectroscopic evaluation of CPC pastes initially and after hardening for 7 days in water

The IR spectrum for hydroxyapatite showed the characteristic bands for calcium phosphate in the interval from 4000 to 400 cm^−1^ (Fig. 4). The OH bands appeared at 3572 cm^−1^ (stretching) and 634 cm^−1^ (vibration). The phosphate bands were the largest, at 1094 cm^−1^ and 1045 cm^−1^. These appeared specifically in the range of 1000 to 1150 cm^−1^ (asymmetric stretching or ν3). A defined band also appeared at 962 cm^−1^ (symmetrical stretching or *v*1), from 560 to 610 cm^−1^ (bending or *v*4) and 479 cm^−1^, showing *v*2 mode. Vibrations related to the organic phase within the cement (oil-surfactant mixture) appeared at 2900–300 cm^−1^ (v(C-H)) and around 1740–1750 cm^−1^ for the carbonylic (vC = O) vibration. Both were reduced in their intensity after hardening, which is a result of an exchange with water by diffusion processes.

### Mechanical evaluation

**Fig. 5:**
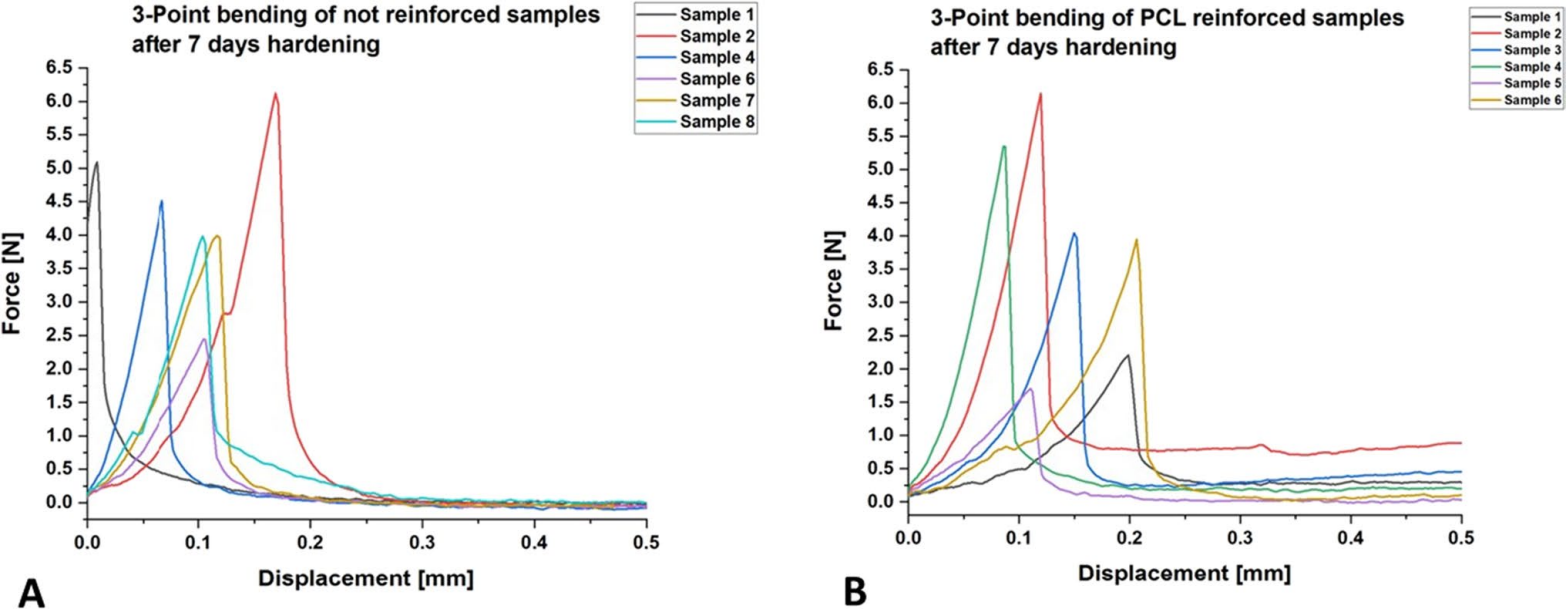
Force displacement curves of CPC samples without reinforcement with PCL fibers (**A**) and with reinforcement with PCL fibers (**B**) after 7 days of hardening in a water bath

A standard 3-point bending experiment has been conducted to identify effects of the incorporated PCL meshes within the cements. All fabricated and tested samples were consistently numbered. While for the PCL-reinforced samples, all specimens 1–6 could be successfully tested, by 3-point-bending, sample numbers 3 and 5 for the non-reinforced laminates broke before testing and had to be replaced by new samples 7 and 8. The results for force displacement are shown in detail in Fig. 5. No significant difference between PCL-reinforced and non-reinforced samples was measured for maximum force (*p* = 0.587) and for sample displacement at maximum force (*p* = 0.167). Neither were there significant differences for maximum stress (*p* = 0.757) or sample displacement at maximum stress (*p* = 0.144). The values for maximum stress and forces are displayed in Table 1. The maximum force that must be applied until the material breaks is slightly higher for non-reinforced samples than for PCL-reinforced samples. With regard to the maximum stress, the value was slightly higher for PCL-reinforced samples than for non-reinforced samples. Displacement at maximum force and stress is both higher for PCL-reinforced cement samples.

**Table 1:** Average values for maximum force and stress load before material fatigue for CPC samples with and without PCL fiber reinforcement

	Not reinforced	PCL-reinforced
Max. force	4.4 ± 1.2 N	3.9 ± 1.7 N
Max. stress	0.27 ± 0.08 MPa	0.28 ± 0.08 MPa
Displacement at maximum force	0.095 ± 0.053 mm	0.140 ± 0.053 mm
Displacement at maximum stress	0.096 ± 0.055 mm	0.144 ± 0.050 mm

## Discussion

Craniofacial defect situations involving bone tissue are definitely not a monomorphic disease pattern. The complex morphology of the involved bones, which is associated with various different functions requires individual approaches for different kinds of defects. Besides the aesthetic reconstruction of affected patients, a functional rehabilitation, as well as the protection of vulnerable structures, must be taken into consideration.

The range of materials which are currently used for bone reconstruction of the skull or have been suggested for this purpose is vast. It comprises autologous transplants as well as alloplastic ones, amongst which the latter can be further divided into resorbable or non-resorbable ones and implants which are prefabricated or have to be modified intraoperatively. Autologous transplants are still regarded as the golden standard of bone reconstruction. Grafts from the calvaria itself, the ribs, or the iliac crest have been reported as suitable for neurocranial reconstruction [23], whereas fibular grafts, iliac crest grafts, or scapular grafts are preferred for the reconstruction of midfacial or oromandibular bone defects [24, 25]. Still, main limitations of all these autologous grafts remain a limited availability and donor site morbidity. Due to this, alloplastic materials have caught more and more attention as bone replacement materials for skull defects. For example, for cranioplasty after decompressive craniectomy, methods currently employed include the fitting of implants made from hydroxyapatite (HA), polymethylmethacrylate (PMMA), porous polyethylene (PPE), and titanium [25]. For the reconstruction of maxillofacial bone defects—besides the aforementioned PMMA, HA, PPE, and titanium implants—the use of calcium phosphate and polyetherketone or combinations of alloplastics respectively alloplastic and autologous bone transplants are described [26]. As it can be seen, the use of cements with different formulations is quite common in skull reconstruction. Nevertheless, according to our knowledge, no other working group tried to perform skull reconstruction with a composite made from polymer fiber mats and cements pastes. Although feasibility of such fiber laminates has already been proven with other basic materials by our working group [22], it has never been applied in a clinical and lifelike context. We believe that, according to the principle of reinforced concrete, the smoothness and the high tensile strength of the PCL meshes [27] combined with the compressive strength [21] of the hardened cements may lead to bone substitutes with very advantageous features.

Due to the fabrication process of the fiber laminates which includes the alternating application of cement-paste and 3D-printed fiber mats layer-by-layer, a basically unlimited number of layers and therefor thickness of the laminates would be feasible. As the fabrication of the single components, especially of the PCL fiber mats is rather time consuming, it seemed worthwhile, to initially fabricate less thick fiber laminates for this study. That is why we decided to use laminates which consisted only of 2 layers of PCL fiber mats. Preliminary studies revealed, that for 3 layers of scaffolds—depending on the fiber spacings of the respective PCL fiber mats—laminate thickness amounted approximately between 0.71 and 1.3 mm. Herein bigger fiber spacings led to less thickness of the laminates [22]. The fiber laminates we obtained measured approximately 2–3 mm of thickness and seemed therefor only suitable in non-load bearing situations where rather thin bony structures had to be replaced (e.g. orbital floor, maxillary sinus, frontal sinus, thin calvaria). Load-bearing situations and replacement of complexly shaped bones do not seem favorable in the first place. Still, the replacement of rather thick but evenly shaped bones such as the calvaria seems possible when the laminate thickness is adjusted by the addition of further layers. In other defect situations where only very delicate bony structures are to be replaced, functional aspects must also be taken into account to a greater extent. For example, in the area of reconstruction of the orbital floor, for which the laminates examined here could be used well in principle, too great a thickness of the laminate can lead to ocular protrusion of the affected side with associated problems such as double vision and strabismus. In the present study, the CPC-PCL laminates could be fitted very well into the defect situation of the orbital floor, however, according to the laminate thickness, an increase of the orbital floor in the area of the caudal orbital rim by approx. 2 mm was shown, which could lead to the above-mentioned problems. This problem could be avoided, where possible in case of more dorsal defects, if the laminates were placed more dorsally from the outset and the floor on which the laminates are fitted (mirrored from the opposite side) was designed virtually deeper in CAD by the amount of the material thickness to compensate for this effect. Besides, for a better stabilization of the laminates in the defect, also overlapping margins (ventral extensions) over the orbital rim would be possible. As the eyeball itself lies behind the caudal orbital rim, the extension of the laminate should not change the position of the eyeball itself and therefor lead to its elevation. However, this type of problem does not occur in areas that have more of a supporting and covering function. These include, to varying degrees, the clavarian and ventral midface defects chosen in this study. In both situations, the defect could be reconstructed well. In the calvaria region, we decided to clamp-fit the laminates due to the defect size in relation to the size of our laminates. This could be realized without any problems, but the question arises whether this would be reasonable to realize in real clinical cases, because there is a certain risk of dislocation of the laminate, especially intracranially. It would make sense to add overlapping structures at the edges of the defect to further secure the position of the laminate. In the other case of a defect of the facial maxillary sinus wall examined here, such overhangs could already be realized. The defect could be completely covered without significant substance application and its position secured at the same time. Due to the complex bone morphology in this area with simultaneously convex and concave bone contours, however, the modeling of the laminates was somewhat more challenging, but ultimately succeeded without leading to material failure.

Nevertheless, clinical handling of the fiber laminates proved to be very suitable. The initially smooth consistency allowed for a detailed adaption of the fiber laminates onto the templates. Greater overlaps could be either removed before hardening with a scalpel or after hardening in case of smaller interferences with a plaster knife. However, the removal of smaller protrusions after curing of the laminates, was in most cases associated with fracturing of the edges. This resulted in fraying and reduced accuracy in some cases. These fraying subsequently diminished the accuracy of fitting of the laminates in the defect situations, which was expressed by smaller gaps between bone and laminate in the margin areas. However, most of these gaps originated in the removal of the laminates from the underlying skull model. This in turn, is a measure, which would not be performed when laminates were applied in vivo. Nevertheless, the adaptation of the laminates to the clinical defect should ideally take place for the most part before curing in order to reduce inaccuracies in the fit. This way, very good fitting and contouring could be achieved. The following fixation on the model skull was initially supposed to be performed via conventional osteosynthesis microplates. As this lead to laminate fracture or fraying of the drill holes on the margins of the fiber laminates, we preferred to fixate the samples via overlapping margins or clamp fit in the clinical defects. This may not guarantee a fixation as stable as osteosynthesis plates would provide, but considering a use of the laminates in rather non-load bearing defects, a rigid fixation does not seem to be mandatory. Furthermore, especially in regions which are either difficult to access or very fragile, such as the facial wall of the maxillary sinus or the orbital floor, osteosynthesis sometimes is not even possible anyhow. Regarding handling properties and fit, the examined fiber laminates were comparable with other prefabricated CAD/CAM bone substitutes like tricalciumphosphate [28], hydroxyapatite [29], or polydioxanone lactide [30].

Hardening of the cement laminates is initiated in the contact with moisture or water, which diffuses into the cement paste starting the typical dissolution—precipitation cement reaction. The structural analysis showed, as expected, that the CPC pastes cured to a solid cement containing HA while the amount of oil-surfactant phase decreased significantly. XRD results suggested that the monetite reacts with the tetracalcium phosphate to form hydroxyapatite, however this reaction was not yet completed after 7 days and is thought to proceed after implantation. These results were similar to what has already been reported for other calcium phosphate containing cement formulations in terms of structural changes within the cement pastes during curing [31]. Carbonyl groups, e.g. aldehydes, esters (mygliol belongs to them), ketones, carboxylic acids, amides, or peptides, each have a strong and well recognizable IR absorption band of the C = O stretching vibration around 1600–1700 cm^−1^. This peak decreased in the IR spectra during curing ex vivo, indicating that the oil percentage decreases in the hardening process, while residues can still be found in the hardened paste. It should also correlate with other signals between approx. 500 cm^−1^ and 1500 cm^−1^, in which case it is more difficult to assign it exactly to individual molecule parts. The remaining organic residues is however uncritical for the cytocompatibility as oil-surfactant mixture consists only of pharmaceutically approved compounds and such premixed CPC pastes are already approved as medical products for bone replacement applications [32]. The final mineral phase of the cements used in the laminates consists of approximately 60% HA, 30% TCP, and 10% dicalcium phosphate anhydrous. Thus, the composition of the mineral phase of the laminates is somewhere similar to clinically used products such as Bone Source™ cement. This type of material is well known in literature as an only slowly degrading biocement with degradation times of more than one year [33]. Degradation of such cements occurs by osteoclastic cells without the formation of toxic products. Nevertheless, the material displays good bonding ability and subsequent healing of the defects [33]. The second component of the laminate is PCL, which is also approved as medical product and also shows only a very slow degradation speed of several years [34]. The acidic by-products formed by the hydrolysis of PCL will be buffered by the calcium phosphate component and hence, a detrimental biological effect during degradation is not expected. Therefore, primary wound healing, which normally takes place within days in the soft tissue area and within weeks in the bony area, should not be influenced by the material combination used, since its degradation takes years and it releases only a few degradation products per time in its course. However, wound healing could be adversely affected by small fragments from the bone graft substitutes breaking off and being carried into the surrounding tissue. Here, the marginal areas are particularly at risk of fracture. In the present work, breakout of the marginal areas was observed when attempting to drill holes for osteosynthesis and also when lifting the laminates from the model surface. This led in each case to incongruities in the defect margin area and insufficient coverage of the defects. Since the resorbability of the inserted grafts in vivo would not require them to be lifted off again, this problem would not arise.

The mechanical evaluation of CPC with or without reinforcement with PCL fibers displayed no significant difference between the samples for maximum force and stress, indicating no reduction of the overall mechanics of the cement while still improving handling of the laminate. Overall, the samples are comparable within their groups, indicating homogeneity within the samples. A complete conversion to stress/strain is difficult from a 3-point bending experiment and is not done in this dataset; an elastic modulus has not been determined. Reinforcement of CPC pastes with smaller fibers has already proven its effectiveness in increasing the mechanical properties of the original cement pastes. Herein, an increasing fiber length led to an enhanced material strength, whereas injectability was reduced [35]. In our study, mechanical properties considering force and stress displacement itself were not improved significantly by fiber reinforcement of PCL fiber mats, whereas they have neither been impaired. However, the fact that two non-fiber-reinforced samples broke during the preparation of the material test, while this did not happen with any of the fiber-reinforced samples, can be seen as a subtle indication of improved stability due to the insertion of fibers. Furthermore, there has been a major improvement of clinical handling properties by combining PCL fiber mats and CPC pastes.

With regard to the material properties of the laminates, further improvements can nevertheless be sought. For example, breakout and fraying of the laminates at the edges proved to be a limiting factor for osteosynthesis. However, the possibility of osteosynthesis in principle would certainly expand the range of applications of such laminates. In the future, an improvement of this material property could be aimed at in this context by modifying either the cement formulation or the fiber density or geometry. Such modifications could also lead to a further improvement in strength, which could, for example, enable the production of thinner laminates to replace gracile structures, such as in the context of orbital floor reconstruction, without clinical disadvantages. Nonetheless, the excellent clinical handling seems to clearly outweigh the laminates’ properties that need improvement as mentioned here. Fiber reinforcement enabled the prefabrication of planar bone replacement materials in order to cover flat defects, which would not have been possible with single components alone.

## Conclusion

The combination of oil-based cement pastes and 3D-printed fiber meshes represents a very promising option for the reconstruction of clinically relevant defects in the field of oral and maxillofacial surgery. In this way, flexible and storable fiber laminates could be produced that combine the advantages of both starting materials, namely the high strength of CPCs with the pliability of PCL fiber mats. The mechanical performance of the cements is not affected by the incorporation of the polymer fibers. Even if fixation by conventional means such as osteosynthesis does not seem feasible in its current form, the use of the fiber laminates presented here in non-load-bearing areas that do not necessarily require rigid fixation can be quite profitable. Further adaptations of both the cement and the fiber components and their combination could provide a remedy for this in the future.

## Data Availability

The data supporting the findings of this study are available on request from the authors.
